# A patient navigation initiative to improve access to breast cancer care in Cali, Colombia

**DOI:** 10.1002/cnr2.1564

**Published:** 2021-11-01

**Authors:** Andres H. Perez‐Bustos, Mavalynne Orozco‐Urdaneta, Raúl Erazo, Patricia Cordoba‐Astudillo, David Gallo, Carlos Muñoz‐Zuluaga, Michelle Sittig, Armando Sardi

**Affiliations:** ^1^ Fundación para la Prevención y Tratamiento del Cáncer Cali Colombia; ^2^ Partners for Cancer Care and Prevention Baltimore Maryland USA; ^3^ The Institute for Cancer Care, Mercy Medical Center Baltimore Maryland USA

**Keywords:** breast cancer, early diagnosis, healthcare disparities, patient navigation

## Abstract

**Background:**

Patient navigation is the logistical and emotional support necessary to achieve diagnostic and treatment compliance. It can improve time to diagnosis, initiation of treatment, and patient satisfaction, as well as reduce the cost of treatment. Colombia has a well‐defined Cancer Control Plan, but its implementation is lacking.

**Aim:**

To implement the first patient navigation initiative in Colombia, as part of a pilot program for the early detection of breast cancer.

**Methods:**

The process involved assessing and addressing the barriers faced by women to access breast health care by providing training for health personnel, strengthening primary health care providers, and coordinating diverse level institutions for the provision of services. This led to the design and implementation of a navigation strategy focused on the needs of patients in Cali, Colombia and the involvement of the local health system to provide such services.

**Results:**

Time to diagnosis was significantly reduced; research advanced by the Colombian National Institute of Cancerology shows that the average time between the first medical consultation and diagnosis was 91 days (CI 95%: 82–97 days), while this study carried out the same process in an average of 30 days, but patients still had issues with continuity of treatment due to financial strain between healthcare providers and insurers. Navigation, however, manages to overcome many of these problems by assisting women in the clinical and administrative care processes and seeking well‐being for the beneficiaries. In addition, patient navigation helped identify critical failures in care, such as fragmentation of care and excessive bureaucracy. The navigation process improved data collection and established agreements to simplify and make the delivery of care more efficient. In addition, it generated partnerships between service providers and insurers.

**Conclusion:**

While several barriers and poor understanding of the navigation process still exist, a navigation program can help implement a Cancer Control Plan.

## 1 BACKGROUND

There is a difference, or at least a distance, between the health services provided and those received by the general population. This difference is associated with social factors that divide the population, making some less likely to access health services when needed. The causes of these health disparities are poverty, culture, and social inequity,^1^, ^2^, ^3^ by addressing one of these fronts cancer survival and quality of life during treatment can improve. In Colombia, there is a disparity in access to health services; specifically for breast cancer^4^ it is related to educational levels, socioeconomic status, and insurance. These topics can be partially solved with the patient navigation strategy.

Patient navigation initiatives are recognized as a strategy to provide logistical and emotional support to patients that contribute positively to adherence and diligence for treatments of chronic and acute diseases. Specifically, time to diagnosis, initiation of treatment, patient satisfaction with received care, and cost‐effectiveness of treatment are improved. It also provides individualized assistance offered to patients, families, and caregivers to help overcome health care system barriers and facilitate timely access to quality medical and psychosocial care.^5^

The Colombian Health System is an appropriate environment to develop navigation programs since its' universal insurance model covers 94% of the population, in two modalities: (1) contributive for employed people with monthly payments based on income, and (2) subsidized for people who can pay a monthly fee to the health system without a stable job or income. The existing Cancer Control Plan 2012–2021^6^ has clear goals focused on quality in breast cancer care and early detection, but it has not been implemented. Additionally, it did not include a patient navigation program. In Colombia, breast cancer is the leading cause of death by cancer in women: the ASR* for breast cancer mortality in Colombia is 13.1, higher than USA (12.4)^7^; nevertheless, it is lower in Colombia than the USA: 48.3 and 90.3, respectively. Colombia has fewer new cases of breast cancer and higher mortality, and most of cases of breast cancer are diagnosed in late stages, in higher proportions in elderly women, with less education in the subsidized health plan.^8^

It is important to mention that there are no time standards for the various health care processes, making it difficult to determine causes for diagnostic delays, and in the same way, there are no previous studies about patient navigation strategies developed in Colombia. This study compares the results at two timepoints (2012 and 2019). According to a study conducted in Bogotá, Colombia the median time between the first consultation and diagnosis was 91 days (CI 95%: 82–97 days) and 30% of women consulted clinical care at least two times for primary symptoms before the diagnostic process was initiated and in 51% of cases, diagnosis was delayed more than 3 months.^9^

In order to promote the timely access to breast cancer diagnosis and treatment, in 2012 the patient navigation program was proposed to a secondary healthcare provider Hospital in Cali† (Colombia) by the Fundación para la Prevención y Tratamiento del Cáncer (FPTC) and Partners for Cancer Care and Prevention (PFCCAP), two sister organizations from Cali, Colombia, South America, and Baltimore, Maryland, USA, respectively, that aim to reduce the mortality of breast and cervical cancers through early detection, disease prevention, and health promotion. Hereinafter, *FPTC* will refer to both organizations.

## 2 AIM

The objective of this study was to create and implement a patient navigation program as a strategy to increase the use of breast screening and adherence to treatment for eventual cancer diagnoses. The hypothesis was that diagnostic interval and treatment times could be shorter if patients had support to face the breast cancer screening and treatment, and that can change the results (stage at diagnostic) in benefit of patients.

This document details the process of implementing the first patient navigation initiative in Colombia as part of a pilot project for the early detection of breast cancer^10^ It describes the barriers and the implementation of a successful patient navigation program.

## 3 METHODOLOGY

In 2012, the Colombian Ministry of Health published the Cancer Control Plan 2012–2021, which described the ideal diagnostic and care processes for cancer treatment but did not indicate how to reorganize the health system to benefit women who already had the disease. FPTC sought the inclusion of key actors to implement the concepts expressed in the aforementioned plan. This project focused on establishing a relationship of trust, teamwork, and quality control, which would eliminate unnecessary administrative procedures and reduce the burden on patients, while optimizing the resources available within the primary healthcare centers. This was accomplished by collaborating with insurers (called Health Benefit Plan Management Companies, EAPB in Spanish), Health Service Providers (IPS in Spanish), laboratories, universities, entrepreneurs, and local politicians.

To counteract time barriers, FPTC financed and implemented a “Breast Cancer Early Detection Pilot Program” in Colombia to be executed with local health providers and an insurer. The process was advanced in four stages, previously planned in coordination with local institutions:

1. Evaluation of barriers, potential allies, and solutions.
2. Training doctors and nurses on comprehensive breast cancer care and strengthening institutions for early diagnosis.
3. Coordination of low and high complexity institutions for timely reference of cases between levels of care.
4. Creation of a strategy for the navigation of patients in diagnostic and treatment processes for breast cancer, adjusted for the needs and possibilities of Cali.

### 3.1 Evaluation of barriers, potential allies, and solutions

In 2012, a secondary healthcare provider was chosen to measure barriers and possible solutions to design the navigation strategy. That same year, a survey was conducted on 105 women with breast cancer who attended a healthcare campaign organized by FPTC. These patients were all diagnosed at advanced stages of the disease and had incomplete chemotherapy treatment plans. In addition, 47.6% of women were still waiting for their first surgery. The majority (68%) of respondents had not completed secondary education, 50% had only primary education, 55% were unemployed, and 28% were under 50 years old. This information indicated the magnitude of the problem, which, in addition to clinical, also included sociocultural issues. Accordingly, the patient navigation strategy aimed to address these specific needs.

The next step was an invitation to key players (primary and secondary healthcare providers, local politicians, entrepreneurs, academics, and the EAPB with the greatest insured population) to discuss breast cancer, existing barriers, and possible solutions. Using a qualitative approach, promoting the situation analysis among a panel of experts, a specific list of administrative barriers associated with case management within the health system was developed (Table 1).

**TABLE 1:** Perceived barriers in the diagnostic process for breast cancer in Cali (2012)

Barriers perceived: healthcare providers	Barriers perceived: insurance companies
Breast cancer screenings in tertiary healthcare providers, saturated by patient volume	Patients diagnosed at advanced stages, increasing costs
Insurers issued authorizations for each procedure (mammography, ultrasound, biopsy, surgery, etc.) and services from multiple providers, fragmenting care, and limiting ability to follow‐up	Health providers did not guarantee care with quality and relevant expenditure: fragmented authorizations allowed for control of procedures and medications, reducing the financial risk of insurers
Lack of breast cancer screening programs and untrained staff to treat breast cancer patients. The insurers, therefore, did not send their patients	Untrained cancer screening personnel, which posed risks to insurers. Patients with advanced disease presented directly to tertiary healthcare providers
The insurers contracted high‐level services with the most economical provider, sacrificing quality, and generating fragmented services	The high volume of patients diagnosed impacted decision‐making without implementation of a cost–benefit strategy or quality control program. Referrals to a tertiary healthcare provider increased providers' delay due to over saturation of centers

In addition, using interviews and thematic analysis of patients' interviews, barriers related to cancer treatment and adherence were identified:

1. Patients were responsible for providing medical history and arranging appointments for treatment and follow‐up of their disease.
2. Lack of coordination between the three levels of health facilities resulted in significant delays in care. When a general practitioner in a local community health center consulted a woman with breast symptoms, referrals are made to a higher level of care, without any connection or contact between those institutions.
3. The care processes (surgery, chemotherapy, etc.) offered by the high complexity health facility were mediated by a complex administrative process called “authorization” which granted payment of these services. During treatment, patients were responsible for managing this authorization process and mobilization between saturated institutions for service requests. In addition, authorizations were required for every procedure, including each chemotherapy cycle.
4. Healthcare providers were not prepared to implement the 10‐year Cancer Control Plan and this was reflected in the low quality of services provided.
5. The patients did not have adequate knowledge about their disease and the complex administrative processes that they had to follow for their treatment in the health system.
6. There were no available cancer staging databases to properly determine treatment plans or aid in appropriate administrative decisions. Staging was not performed for most patients, even those actively receiving treatment.

By analyzing the characteristics of patients surveyed and taking stock of the barriers experienced by users of the health system, the FPTC proposed patient navigation as a strategy to improve the quality of care for a specific group of diagnosed women: those insured by the EAPB interested and residing in the west‐side of Cali. The pilot study was implemented in August 2012 and concluded December 2019.

The primary healthcare providers were selected for reasons associated with operational capacity (health facilities and a hospital, all in areas of high population density, and located within the neighborhoods of Cali's west‐side and north‐east), stable financial situation, commitment of managers, and ease of access by potential users. Two principles of the project were also established: (1) focus primarily on early detection, and (2) a screening program would not be available until diagnostic technology and quality could be guaranteed.

### 3.2 Training doctors and nurses for comprehensive breast cancer care and strengthening institutions for early diagnosis

After allies and needs were identified, the next step was to train general practitioners and nurses on the correct technique to perform a clinical breast exam and then incorporate it as a routine procedure, during any medical consultation by local healthcare providers. Thus, staff was able to follow the Clinical Practice Guides^7^ and Integral Health Care Routes^11^ issued by the Colombian Ministry of Health for breast cancer. Subsequently, medical personnel with a specialty in radiology, contracted by FPTC, provided advice, education, training, and constant updating of the local medical team. Finally, a monthly virtual training schedule was created to provide education to primary physicians and nurses on breast cancer early detection which was initiated in January 2013. All training was guided by the National Cancer Control Plan and financed by the FPTC.

After training healthcare personnel, the guideline for early detection was adopted to include all women between 50 and 69 years of age and any woman with a history of breast cancer or with an abnormal clinical breast exam. FPTC financed 50% of the cost of digital mammogram equipment that was installed in a low complexity health care facility in 2015. The financial donation was accompanied with training for radiologists and radiology technologists to ensure quality in performing and reading of mammography.

### 3.3 Coordination of primary and complementary health facilities for the timely transfer of cases between levels of care

Colombia provides health care coverage for more than 94% of its population. This made it possible to create early detection programs or timely breast cancer care in a wide network of healthcare providers since the economic burden does not fall on the patient and family, but on insurers who receive funds from the Colombian health system. In this phase, conditions were created to develop the pilot program and the patient navigation strategy, counteracting the fragmented health system.

The insurer and FPTC collaborated to develop the project roadmap. Primary health providers would perform mammography and refer cases with abnormal results to one of the participating complementary health providers with cancer services. The conditions included quick management when receiving cases, sharing medical history directly between providers, communication between treating physicians, and reducing administrative procedures that previously complicated access to health services.

To implement the project and coordinate management between health providers and insurers, the concept of patient navigation was proposed, which included workshops and meetings among participating organizations.

Protocols for improving the diagnostic process were constantly adjusted, seeking to reduce administrative burden, simplify bureaucracy, and change patient interventions to direct management between institutions. The patient navigation strategy managed cases within the complementary IPS and promoted the creation of contracts between institutions designed for the well‐being of patients through the program manager. Table 2 describes the activities of the pilot program participants.

**TABLE 2:** Activities framed in the navigation strategy

Responsible	Activity	Objective
Program manager	Adjustment of the treatment pathway	Adapt the alliances between IPS and EAPB to facilitate the care process for female users Continuous evaluation of the evolution of cases
General physician	Description of family cancer history, significant consultation findings (excessive stress, panic, no support network)	Update the database with information that describes the specific needs of the patient
Patient navigator (internal)	Patient coordination after initial consultation with review of personal/clinical data	Patients change address and contact number frequently, so they are asked to provide additional contact information
	Identification of individual barriers to access health	Checklist of common barriers: Insurance Knowledge of the insurer's route of care and allied lenders Identify patient support network Employment status Cultural or religious limitations Physical limitations Potential impact of out‐of‐pocket spending Fear or anxiety during the process Understanding and empowering the treatment process
	Develop a plan to solve identified barriers	Solve the resource barriers (not only economic) for the patient: support networks, couples, health insurance, social groups, and so on
External navigator	Follow‐up of cases related to Complementary IPS	The navigation team monitors the patient through constant communication with the user and monitors the evolution of the administrative procedures of the case through contacts within the Supplementary Provider. Assistance is provided if barriers to healthcare access is identified
	Management of cases and potential solutions for/with complementary health providers	When barriers are contractual between provider and insurer or for lack of medicines, the navigator provides help to request transfer to another provider. This involves constant monitoring to avoid delays in treatments

### 3.4 Navigation strategy for Cali

Patient navigation was a method implemented coordinating health providers and insurers to benefit patients through promoting timely attention and treatment adherence. The lack of trained personnel motivated the FPTC to bring a patient navigation expert from the United States based *New Life* organization, to Cali, Colombia.^8^ Trainings were carried out in two locations. The first at a primary health facility, which initiated the process by training its general practitioners to detect abnormalities through clinical breast examinations and training doctors, nurses, nursing assistants, psychologists, social workers, and administrative staff on the basics of navigation. The second occurred at the Universidad del Valle School of Nursing, where 130 nursing students were trained. A patient navigator position was funded to coordinate the program and serve as a contact within the complementary health providers. The navigator helped generate a network that would provide diagnosis specific treatment within the pilot program.

Implementing the aforementioned steps in the primary health providers allowed for early detection of breast cancer through mammogram screenings by a qualified provider located geographically close to patients. It was also the location where general practitioners were providing their year of required social service. The purpose of the tertiary healthcare providers was to expedite patient access to treatments (surgery, chemotherapy, and radiation therapy), in addition to familiarizing future nursing professionals, from the largest university in southwestern Colombia, with the navigation strategy.

Additionally, two FPTC team members were trained in patient navigation by the American Cancer Society (ACS) at Mercy Medical Center in Baltimore. The Cali navigation program had to adapt to both local barriers and to the Colombian Health System. Identified barriers included previous clinical guidelines, locations of performed procedures, providers delay, establishing roles and jobs within the system, and administrative constraints. The patient navigation program was constantly updated, resulting in a network of key contacts within the city's health providers with access to the institutional protocols making the administrative experience easier for patients. The ACS‐trained navigation team replicated training in Cali and two subsets of the navigation program were created: an internal navigation program, which supported all health services and administrative processes that occurred at the primary health facility and an external navigation program that supported all processes at outside health institutions.

Patient navigation begins by identifying women able to have screening mammograms or breast ultrasounds. Through the insurer's databases and community outreach, identified women with limitations to access to screening are supported. Patient data were recorded in a database shared among the program staff. Case tracking was done by telephone with weekly calls tracking progress. These calls were prioritized based on the need for diagnostic testing (Breast Imaging Reporting and Data Scores [BIRADS] 0, 3, and 4) and those diagnosed with breast cancer. Women requiring repeat mammograms were contacted directly.

The objective of FPTC was to serve as a bridge between institutions and seek the coordination of health service facilities and insurers, prioritizing the quality care for early diagnosis and treatment. This nongovernmental organization (NGO) should be eliminated from the care scheme as soon as the patient navigation services are integrated into the dynamics of health care providers. To assist in achieving this goal, FPTC promoted the creation of *Breast Health Programs*, included the patient navigation strategy that develops specific indicators, ongoing training, assigned personnel for clinical care, and administrative management.

## 4 RESULTS

The patient navigation strategy improved the timing of care for diagnosed patients. Research advanced by the Colombian Institute of Oncology in 2011 shows that the average time between the first medical consultation and diagnosis is 91 days (CI 95%: 82–97 days),^5^ while this study carried out the same process in 27 days due to reduced time spent at each interval within the care schedule (Table 3).

**TABLE 3:** Change time to care, before and after the implementation of the navigation program

Interval (days)	2012 (105 cases)	2019 (51 cases)
First medical appointment → Biopsy	65	24
Biopsy → Diagnosis	33	3
Diagnosis → Oncologist appointment	28	18
Oncologist appointment → First chemotherapy session	87	15
Oncologist appointment → Surgery	93	30
First chemotherapy cycle → Secondary chemotherapy cycle	57	24

Prioritization was a constraint due to limited resources and the search for optimization‐sustainability of the strategy. According to the Cancer Control Plan, the patient navigation strategy was established for patients with BIRADS 0, 3, and 4 screening mammogram scores and for all patients classified as the uninsured poor population (Table 4).

**TABLE 4:** Mammography screening performed at primary healthcare provider

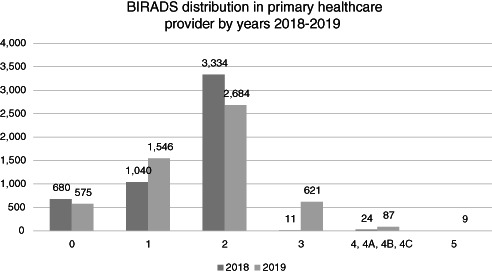

At the start of the project, an authorization was required for each step of the clinical pathway and managed by the patient. This includes collecting the sample obtained in the breast biopsy, delivering it to the laboratory for processing, recollecting the samples with results, and delivering them back to the physician with additional wait times to discuss diagnostic results. At each location, the patient had to carry specific documentation (copies of the identity document, medical history, pathology order and results, and a document exonerating responsibility) without understanding the situation. As shown in Table 3, in 2012 the average time between biopsy and diagnosis was 33 days. With the introduction of the patient navigation strategy and assistance in transporting samples, the interval was reduced to 3 days. In the cases of advanced age, physical limitations (i.e., blindness), absence of social support network, or others, assistance was also provided.

Another result of the implementation of the navigation strategy was the ability for patient follow up after the screening or diagnosis process. In Colombia, there is not yet a single database for health system users, nor are there unified records in each IPS and EAPB. The database is managed by the navigation leader at the IPS Primary program, adjusted among the administrative team of the primary healthcare provider and was advised by FPTC. The data collected are listed in Table 5.

**TABLE 5:** Variables collected by health service providers

*Demographics*			
Name	Identity card	Age
Phone	Address	EAPB insurer	
Care site	Health network	
*Clinical data*			
Diagnosis	Tumor staging	Vital status	
Observations	Consultation with primary IPS		
*Diagnostic testing*			
Test performed	Order date	Date of test	
Time to results (days)			
*Medical*/*radiation oncology*			
Referral to medical oncology	Time to consultation	Treatment start date	
Time to treatment (in days)			
*Surgery*			
Surgery order date	Time to consultation	Surgery date	
Time to surgery (days)			
*Follow‐up*			
Treatment follow‐up	Update information (new medical appointments, new barriers, reiterative barriers, etc.)	Keep the screening process by cell phone reminders and medical appointment scheduling	

To counteract delays in the provision of services, legal or administrative measures had to be used to guarantee the care processes for women diagnosed with breast cancer. Since 2015, two women have received legal advice and counsel in the structuring, presentation, and follow‐up of guardianships against the insurer so that they can continue their treatments. In both cases, the judge ruled in favor of the patient and treatments were restarted. Both women managed to finish their treatments, albeit with delays and interruptions, and are currently alive. In the same period, support has been requested for 11 women before the Superintendent of Health, a state administrative body that supports these cases. In all cases, the EAPB responded, and treatments continued.

A common barrier is the patients' inexperience with the diagnostic‐treatment process and the bureaucracy that must be faced to access the health system. Since 2017, the process of internal authorizations has been routine. This simplified the work of the navigation team, but it also created a new challenge in that women no longer had physical proof of the orders issued making it difficult to calculate the appropriate timing for treatments. It was difficult to establish a case for a delay in medical care.

## 5 DISCUSSION

The implementation of a patient navigation program had a positive and direct effect on reducing time to diagnostic and medical care for women with breast cancer. It was an effective strategy to implement the guidelines published by the Colombian Ministry of Health and coordinate roles. It was also effective in mitigating the fragmentation of the healthcare system through direct support for women by explaining the process, providing continual guidance, and intervening with providers to facilitate services.

However, there are barriers, such as political dynamics, that cannot be resolved by the patient navigation strategy. In Colombia, the government cabinet election takes place every 4 years, involving a change of administrative and assistant staff. Thus, each election involves a restructuring the breast cancer screening programs and patient navigation strategies. The new political administrations designate new leaders who are unaware of the processes carried out by the previous administration. FPTC is an organization without political affinities, which, since the beginning of its operation in 2012, has passed three electoral processes and the navigation program was reestablished after each.

Patient navigation implementation should be further explored in Colombia through the different levels of care and participants that make up the health system. Barriers to healthcare access and disparity in patient care by insurance type and geographic location (i.e., women with lower incomes and educational levels have subsidized insurance and live further from treatment centers)^10^ may require specific actions to ensure the use of health services. One lesson learned is that secondary healthcare providers centers are not suitable for breast cancer treatment since they do not have the physical infrastructure or staff, nor the execution of the treatment based on existing clinical standards. Organizationally, they had the ability to run a community‐friendly cancer‐screening program that referred newly diagnosed cancer patients to tertiary healthcare providers, reducing system overload, however, the facilities must be geographically close to communities. Compliance with the Cancer Control Plan requires the organization of healthcare services (primary health providers screening focused on early detection of breast cancer and timely treatment in complementary health providers) around the well‐being of women, providing support in the care processes with the patient navigation strategy, and continuous training of health personnel to ensure the quality of the services (training in the health care and university settings).^12^

Another difficulty that has presented itself is the assignment of staff for navigation functions. The role of navigator has not yet been included within the local healthcare centers; thus, the responsibility is usually delegated to a nurse who already has a fulltime workload.

FPTC navigators covered the full workload of the role until administration understood the value of the patient navigation strategy to increase the number of breast screenings and women diagnosed and in treatment for breast cancer, both indicators of quality presented to EAPB to negotiate payments. In this experience, the patient navigators were adopted as a local initiative after a year of support by FPTC. Nevertheless, the lack of training on the patient navigation concept, objectives, and capabilities for medical, paramedical, nursing personnel, and administrative staff remains as a challenge if the navigation strategy is to be expanded to new health care facilities.

Finally, with the navigation system that was implemented, barriers associated with patient and diagnostic delays were broken. Additional coordination, understanding, and follow‐up is needed between primary, secondary, and tertiary healthcare providers in pursuit of universally available, quality treatments. Even with the decreased time to diagnosis, the administrative delays by insurers to deliver the necessary treatment is not at the level required by Colombia's Cancer Control Plan, which on paper is excellent, but is difficult to implement. Quality control and cost‐effectiveness assessments are vital to be able to provide the required services and increase early cancer diagnosis.

Using this experience, it was possible to visualize the key factors that contributed to the large global differences in mortality and survival in cancer: between high‐income and low‐middle‐income countries. Colombia demonstrated a high percentage of cases diagnosed in advanced stages, limited access to treatments by providers with high quality oncology training, fragmented health systems and major coordination problems, as well as sociocultural barriers.^11^ Patient navigation focused on women at higher risk of delays in cancer care to resolve barriers to timely care and contribute significantly to improving patient outcomes. Several studies have shown increased acceptance and adherence to cancer screening, better opportunities leading to a more timely diagnosis, higher rates in completing therapies, and medical appointment assistance, which all benefit patient care.^13^

Patient navigation was effective in addressing barriers and promoting timely access to health services for breast cancer care. No woman should have to go through the experience alone. The patient navigator served as a guide from diagnosis to completion of treatment. The navigation strategy implemented in Cali compensated for the deficiencies that lead to disparities in healthcare. The population which benefited the most were those among low socioeconomic levels with low educational levels and few contacts. Patient navigators managed the conditions of these patients, improved the patient experience, assisting patients through the health system with fewer delays. It is recommended that patient navigation services be included as best practices in comprehensive cancer care in Colombia.

Nevertheless, the sustainability of the patient navigation in Cali relies on the will of each health facility director, related with the level of commitment with the Cancer Control Plan and the patient's wellness. The patient navigation strategy in Colombia does not have the resources: they are not mentioned in health plan, and do not exist in the list of health services paid by insurers. Our experience was possible due to financial support provided by private donors. The institutions interested in patient navigation for this study understood the strategy as an opportunity to increase the number of women screened and improve the quality of services provided, both covered and paid for insurers. The positive impact generated from the experiences shared among participants of the patient navigation program carries the ultimate motivation to continue to improve health services for Colombian women.

## CONFLICT OF INTEREST

None of the authors declares any conflict of interest.

## AUTHOR CONTRIBUTIONS

All authors had full access to the data in the study and take responsibility for the integrity of the data and the accuracy of the data analysis. *Conceptualization*, A.H.P.‐B., M.O.‐U., P.C.‐A., A.S.; *Methodology*, A.H.P.‐B., M.O.‐U., R.E., P.C.‐A., D.G., C.M.‐Z., A.S.; *Investigation*, A.H.P.‐B., P.C.‐A., D.G.; *Formal Analysis*, A.H.P.‐B., C.M.‐Z.; *Resources*, F.M.L.; *Writing—Original Draft*, A.H.P.‐B., M.O.‐U., R.E., P.C.‐A., A.S.; *Writing—Review & Editing*, A.H.P.‐B., R.E., P.C.‐A., D.G., C.M.‐Z., M.S., A.S.; *Visualization*, A.H.P.‐B., C.M.‐Z.; *Supervision*, A.H.P.‐B., M.O.‐U., A.S.; *Funding Acquisition*, M.O.‐U., A.S.; *Data Curation*, A.H.P.‐B., C.M.‐Z.; *Project Administration*, M.O.‐U.; *Validation*, R.E.

## ETHICAL STATEMENT

The institutions involved in the project: *Hospital San Juan de Dios*, *Red de Salud del Norte Empresa Social del Estado* and *Red de Salud Ladera Empresa Social del Estado* approved the development of the project in their facilities. All the patients signed an informed consent to be part of the project.

## Data Availability

The datasets generated during and analyzed for the current manuscript are available from the corresponding author, upon reasonable request.

## Endnotes

* Age standardized rate.

† Cali is the third biggest city in Colombia, the reference for cancer treatment in the southwest of Colombia with available services for breast cancer diagnosis and treatment.
